# Identification of Conserved and Novel microRNAs in Cashmere Goat Skin by Deep Sequencing

**DOI:** 10.1371/journal.pone.0050001

**Published:** 2012-12-07

**Authors:** Zhihong Liu, Hongmei Xiao, Huipeng Li, Yanhong Zhao, Shuangying Lai, Xinlei Yu, Ting Cai, Chenguang Du, Wenguang Zhang, Jinquan Li

**Affiliations:** 1 Inner Mongolia Key Laboratory of Animal Genetics, Breeding and Reproduction, Department of Animal Science, Inner Mongolia Agricultural University, Hohhot, Inner Mongolia, China; 2 College of Life Science, Inner Mongolia Agricultural University, Hohhot, Inner Mongolia, China; 3 Vocational and technical college, Inner Mongolia Agricultural University, Hohhot, Inner Mongolia, China; Virginia Commonwealth University, United States of America

## Abstract

MicroRNAs (miRNAs) are a class of small RNAs that play significant roles in regulating the expression of the post-transcriptional skin and hair follicle gene. In recent years, extensive studies on these microRNAs have been carried out in mammals such as mice, rats, pigs and cattle. By comparison, the number of microRNAs that have been identified in goats is relatively low; and in particular, the miRNAs associated with the processes of skin and hair follicle development remain largely unknown. In this study, areas of skin where the cashmere grows in anagen were sampled. A total of 10,943,292 reads were obtained using Solexa sequencing, a high-throughput sequencing technology. From 10,644,467 reads, we identified 3,381 distinct reads and after applying the classification statistics we obtained 316 miRNAs. Among them, using conservative identification, we found that 68 miRNAs (55 of these are confirmed to match known sheep and goat miRNAs in miRBase ) are conserved in goat and have been reported in NCBI; the remaining 248 miRNA were conserved in other species but have not been reported in goat. Furthermore, we identified 22 novel miRNAs. Both the known and novel miRNAs were confirmed by a second sequencing using the same method as was used in the first. This study confirmed the authenticity of 316 known miRNAs and the discovery of 22 novel miRNAs in goat. We found that the miRNAs that were co-expressed in goat and sheep were located in the same region of the respective chromosomes and may play an essential role in skin and follicle development. Identificaton of novel miRNAs resulted in significant enrichment of the repertoire of goat miRNAs.

## Introduction

MicroRNAs (miRNAs) are significant factors in the regulation of post-transcriptional gene expression and are involved in a variety of important physiological processes such as development, metabolism, and occurrence of disease [1]. MiRNAs are highly conserved across species. They are expressed in specific tissues at particular developmental stages and have been found in plants, animals, and fungi [2]. A series of studies have shown that miRNAs play important roles in the process of cell growth and apoptosis, blood cell differentiation, homeobox gene regulation, the polarity of neurons, insulin secretion, brain morphogenesis, heart development and in the latter stages of embryonic development. For example, miR-273 and the lys-6 miRNA have been shown to be involved in the development of the nervous system in nematode worm [3]; miR-430 was reported to regulate the brain development of zebrafish [4]; miR-181 controlled the differentiation of mammalian blood cell to B cells [5]; miR-375 regulated mammalian islet cell growth and insulin secretion [6]; miR-143 played a role in adipocyte differentiation [7]; miR-196 was found to be involved in the formation of mammalian limbs [8]; and miR-1 was implicated in cardiac development [9]. In another study, miRNAs in the nervous system of the developing cerebral cortex were found to be regulated by the timing, suggesting that they may control the translation and regionalization of miRNAs [10].

Goat miRNAs are under-reported (miRBase, version 18.0, http://www.mirbase.org/cgi-bin/browse.pl) compared with those of some other mammal; 1,572 from human, 662 from mouse, and 55 from sheep. Taking advantage of gene chip technology, quantitative PCR, and in situ hybridization techniques, the expression profiles of miRNAs have been reported in skin development processes in a number of mammalian species [11],[12],[13],[14],[15]. A small RNA (sRNA) library of fetal skin and hair follicles in mice was constructed by Yi et al. [16]. Cloning and sequencing of the miRNAs in the library revealed that it consisted of 91% sRNA in skin and 63% sRNA in hair follicle; the remaining components were degraded RNA fragments [16]. Andl et al. [17] observed that mouse hair follicles were deformed because a deficiency of the dicer had caused the sRNA to remain unexpressed. The deletion of the critical signaling molecules Shh and Notch1 caused follicular dysplasia, aplasia and dislocation of development in the mice 7 days after birth [17]. The phenotypic changes that were observed in hair follicle development confirmed the important role that miRNAs have in maintaining the morphogenesis of animal fur. This growing body of evidence has shown that miRNAs play a critical role in animal fur development and in the morphogenetic processes.

To study the function of miRNAs in skin follicles, we explored goat miRNAs profiles obtained using a high-throughput sequencing technology. We identified conserved and novel miRNAs in the skin and predicted the candidate target genes of novel miRNAs by analyzing miRNA families. The in-depth sequencing and analysis of the goat skin miRNAs reported here will form a solid foundation for the further exploration of their roles in regulation of the hair growth cycle and for the production of goats with increased economic value, including even possible transgenic animals.

## Results and Discussion

### RNA extraction and sequencing

A total of 10,943,292 reads were obtained by sequencing. After removing low quality reads including 3′ adapter reads and 5′ adapter contaminants, 10,644,467 reads, accounting for 99.04% of the original data, were obtained with 18–30 nt in length.. Then, All solexa reads were aligned against the Bos Taurus genome of Ensembl(version 68), using SOAP and 7,273,139 reads were matched to the cattle genome, representing 68.33% of the total reads. (Information S5). Analysis of the sequencing data revealed that non-protein-coding RNA accounted for 22.28% of total reads; however, 5.24% sRNA matched protein-coding genes. The number of known and novel miRNAs was 3,381, accounting for 0.70% and the 6,113,553 reads account for 57.43% of the total reads. Excluding the Nonprotein-coding RNAs, all the other sequences can be treated as miRNAs to analyse.(Table S1).

The sizes of the goat sRNAs were not evenly distributed; most of them (88.89%) were 20–24 bp sequences and the 22 bp sequences alone accounted for 42.30% of the total reads. The next larger fractions were the 23 bp (13.85%), 21 bp (13.21%) and 20 bp (16.40%) sequences. A similar size distribution was observed for mammalian miRNAs for which 22 bp sequences formed the largest group. Similarly, this result was consistent with Bos taurus miRNAs (662 sequences) in mirbase. In Bos Taurus, The majority of the reads were in the range of 20 to24 nt in length, with 22 nts (40.53%) being the most represented class. (Figure S1)

### Identification of conserved goat miRNAs

Many miRNA are conservative across species; there are also numerous species-specific miRNAs. These miRNAs are relatively “young” in evolution, and play important roles in thread development of specific species [18],[19],[20],[21],[22].

Because there are no goat miRNAs in miRbase version 18.0 (http://www.mirbase.org/index.shtml), we compared our sequences with the known cattle and sheep sequences. By blastn against the known and their precursor sequences, there are 3,381 distinct reads with about 10.6 M sequencing counts matched 316 known miRNAs. Additionally, using conservative identification, we found that 68 miRNAs (55 of these are confirmed to match known sheep and goat miRNAs in miRBase ) are conserved in goat and have been reported in NCBI, (Information S1); 248 miRNA were conserved in other species but have not been reported in goat. (Information S2).

Using gene sequence comparisons and expression analysis, Lee and Ambros [23] reported that about 12% of miRNA in C. elegans, Drosophila and some plants are conserved and of these, miR-21, miR-234, miR-260 and miR-287 are highly conserved [23]. We matched our sequences against some of the mammal genomes in miRBase including the human, chimpanzee, rat, mouse, monkey, pig, horse, and cattle miRNAs. By comparing with sheep miRNA sequences, we found that 5 miRNA families containing miR-127, miR-136, miR-154, miR-229 and miR-379, were conserved in all these species It is, therefore, tempting to speculated that these 5 miRNA families are critical in mammal development.

These five miRNA families as well as miR-432 family have been found in these domestic animals cattle, horse, pig, sheep and goat. Mir-432 is known to exhibit relatively abundant expression patterns that decrease in adulthood [24]. We speculated that the expression of mir-432 in goat skin may play a role in the development of the skin and hair follicle.

Genomic sequence data for goat is still very limited, however, the high genetic homology (90%) between sheep and goat has been widely recognized [25].

Taking advantage of the close homology between them, we compared 21 miRNA families in sheep and goat (Table S2), and found that the miR-368 and miR-668 families were expressed only in sheep and goat and not in cattle, horse, and pig. The over-expression of mir-668 increased the activity of β-galactosidase associated with aging, suggesting that mir-668 is an aging-induced miRNA [26]. It is possible that mir-368 and mir-668 may be related to the development and normal apoptosis of skin hair follicle and, therefore, these miRNAs may be involved in regulating the development of the skin and hair follicle.

MiRNA genes are often present in clusters on the chromosomes; clustered miRNAs are expressed cooperatively [27], [28]. Recent studies showed that 42% of human miRNA genes and 50% of Drosophila miRNA genes were clustered [29], [30]. Because the genomes of sheep and goat are incomplete, we located the 21 co-expressed miRNAs on the completed cattle genome and found that, apart from the miR-668 family which is not expressed in cattle, the other 20 families were all clustered in the 66039214–67786949 region of bovine chromosome 21. A further investigation revealed that these miRNAs are also located in clusters on the human and rat chromosomes.

The largest number of sequences (14,461) in our goat dataset belonged to the miR-379 family which was one of the 21 co-expressed miRNAs families. In rats, miR-379 is specifically expressed in the brain where it is thought to regulate the transcription of miRNA genes [31]. We found that it was highly expressed in the skin of goat, indicating that the miR-379 family may play an important role in the transcriptional regulation of skin hair follicle genes. Most reads (highest expression) were from the miR-379 family; next was miR-127 family with 5,235 reads. The miR-127 family has been reported to play a role in regulating the expression of the tumor suppressor gene BCL6, in cell proliferation, and in apoptosis [32]. Another study found that the miR-127 family plays an important role in fetal lung development [33]. We suggest that miR-127 may play a part in skin follicle cell apoptosis and proliferation. Other families that had a high abundance of reads were miR-134, miR-136, miR-154, miR-370, miR-412, miR-431, miR-432, miR-433, miR-485, miR-493, miR-541; a total of 11 miRNA families. The remaining miRNAs were represented by less than 10 reads and were considered to have low expression profiles in the samples (Figure S2). The abundance of miRNAs in our dataset varied drastically. In the goat sequence dataset, some miRNAs were represented by only a few reads while others were represented by thousands of reads. This range in abundance, indicated that many physiological and biochemical processes were being carried out and that the goat contained a large and diverse sRNA population at the growth stage of hair.

The largest miRNA family was the miR-154 family with 18 family members, next was the miR-379 family with six family members, then the miR-368 family with five family members, and finally the miR-329 family with four family members; other families had only one family member).

### Identification of novel goat miRNAs

Predictions of the characteristic hairpin structure of the miRNA precursor have been used to predict novel miRNA; however, it is still very challenging to identify and validate novel miRNAs. Twenty-two candidate novel miRNA which had not been reported in other species were predicted.These candidate miRNAs had expression level represented by from 5–1,408 reads; chi-mir-m22 had the highest expression (1,408 reads). The total number of reads for the 22 candidate miRNAs was 3,384 (Table S3). (Information S3 and S4)

## Experiments

### Tissue samples

The goats were housed at the Aerbasi White Cashmere Goat Breeding Farm in Inner Mongolia, China. In order to get the well-rounded information of miRNA in Cashmere goat skin, three 1-year-old goats (one male and two female) in the condition of natural feed were used. The three skin samples were collected from the lateral body of three Inner Mongolian cashmere goats in October 2010 during the growth (anagen) phases of hair growth., then Stored in liquid nitrogen.

### RNA extraction and detection

Total RNA was extracted using Trizol (TaKaRa, Inc. Dalian, China), phenol-chloroform (Tiangen, Beijing, China) extraction. The RNA samples were dissolved in RNase-free water and stored at −80°C. Before use, the samples were melted on ice, centrifuged and mixed thoroughly. 1 µL samples were denatured at 70°C for 2 min.The concentration of tested samples (272 ng/µL), RIN values(8.8) and 28S:18S ratios (1.6) were measured by Agilent 2100 to identify the integrity of the RNA. We mixed the three samples into one sample with the same concentration, and then took sample of 10 Ug twice for further sequencing.

### *High-throughput sequencing*

Solexa deep sequencing was performed in Shenzhen Genomics, China. After removing the adapter, contaminated sequences, and low quality sequences, clean sequences were obtained. We then conducted a statistical analysis of the distribution of sequence length. The mRNA expression patterns in each of the skin tissues were acquired. The sRNAs that were obtained were searched against the sequences in the Rfam (version 9.1) database (http://rfam.sanger.ac.uk/). As far as possible, non-miRNA sequences (rRNA, tRNA, snoRNA, snRNA and sc RNA) were filtered out. And the sRNA fragments degraded from mRNA were identified by comparing them with the exons and introns of the mRNA. Then, Summarize all alignments and annotation.

### Family analysis of conserved miRNAs

Because miRNAs are conserved between species [34], we used the family classification of the goat miRNAs to estimate the expression profiles of each family (based on the number of reads in each family) after aggregating the conserved miRNAs appropriately. The conserved family and specific family could be verified by comparing the goat reads with the cattle and sheep families. MiRNAs gene families are sometimes expressed as polycistronic clusters [30] that use the same promoter expressed into polycistron and involving coordinate regulation of the developmental processes [35]. It has been estimated that, in humans about one-third of the miRNAs are expressed in this form [34]. We examined the arrangement of miRNA family clusters in the cattle chromosomes,obtaining the results of cluster analysis by the specific miRNA family comparisons.

### Novel miRNAs prediction

The stem-loop hairpin structures of the precursor miRNA of the novel goat miRNAs were predicted using Mireap. Through the interception of a certain length of the genome sequence, to explore the secondary structure and the information of Dicer cleavage sites, energy and other features, we obtained the information of expression profiles of novel miRNAs and predicted the tem-loop structure of candidate miRNA precursors [36], [37]. Although the characteristic hairpin structure of miRNA precursor could be used to predict novel miRNA, it is very challenging to define novel miRNAs. According to the landmark hairpin structure of miRNA precursor, we choose the uncommented sRNA, by comparing with the cattle genome sequence to cut out a certain length sRNA. We use The minimum folding free energy index (MFEI, minimal folding free energy index) which are proposed by Zhang, taking into account of the length of sequences, and set the value of MFEI 0.85 which is difference from miRNA and other types RNA, by Mireap to target the novel miRNA [38].

## Supporting Information

Figure S1 **Length distribution and abundance of the goat sRNA sequences.**
(TIF)

Figure S2 **The abundance of the conserved miRNAs in goat.** Most of the miRNAs were sequenced only a few times, whereas miR-127, miR-154 and miR-375 were sequenced thousands of times. Goat contains a large and diverse sRNA population at the hair growth stage.
(TIF)

Table S1 **Summary of read signatures that match various RNAs.**
(DOC)

Table S2 **Conserved miRNA family analysis.** “−” indicates no expression, “+” indicates expression.
(DOC)

Table S3 **Novel miRNAs analysis.** As shown in table S3, we attained the result by comparing with the bovine genome information. mfe is the minimum folding free energy index.
(DOC)

Information S1 **68 conserved miRNAs in goat.** Description of data: These miRNAs are conserved in goat and have been reported in NCBI.
(DOC)

Information S2 **Novel miRNAs identified in the present study.** Description of data: 248 miRNA sequences were conserved in other species and have not been reported in goat.
(DOC)

Information S3 **Secondary structures of the precursors of the novel goat miRNAs.** Description of data: This file shows detailed information of secondary structures of the precursors of the novel goat miRNAs.
(DOC)

Information S4 **Predicted targets for the novel goat miRNAs.** Description of data: This file shows detailed information of each miRNA hairpin.
(DOC)

Information S5 **Submitted data.** Description of data: We have submitted data in MIAME-compliant format in Gene Expression Omnibus (NCBI).
(DOC)
