# Supplementary figures and images for: Identification of Conserved and Novel microRNAs in Cashmere Goat Skin by Deep Sequencing
Source: PLoS One. 2012 Dec 7;7(12):e50001. doi: 10.1371/journal.pone.0050001 (PMC3517574; doi:10.1371/journal.pone.0050001)

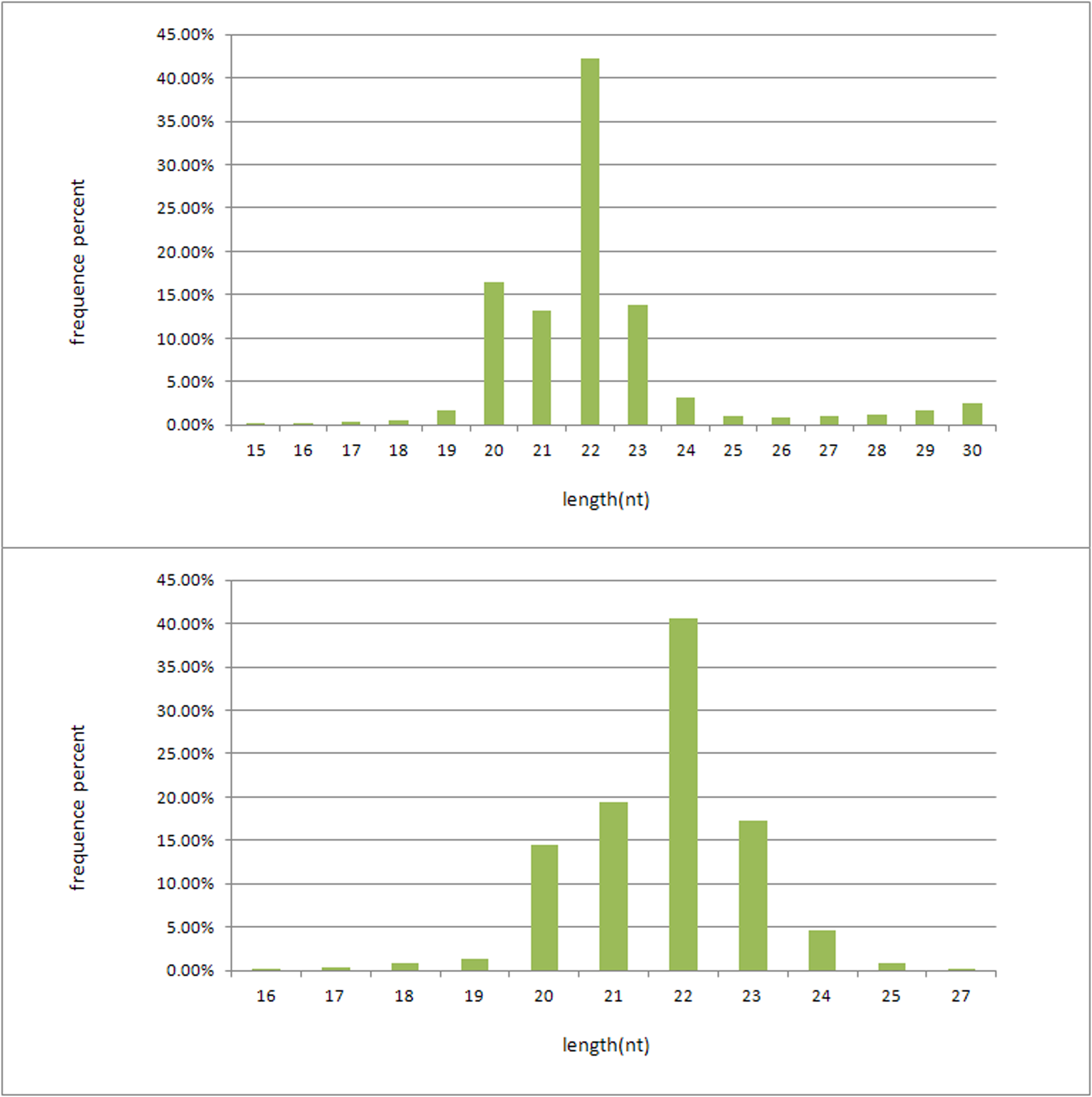

Supplement: Figure S1 — Length distribution and abundance of the goat sRNA sequences. (TIF) [file pone.0050001.s001.tif]

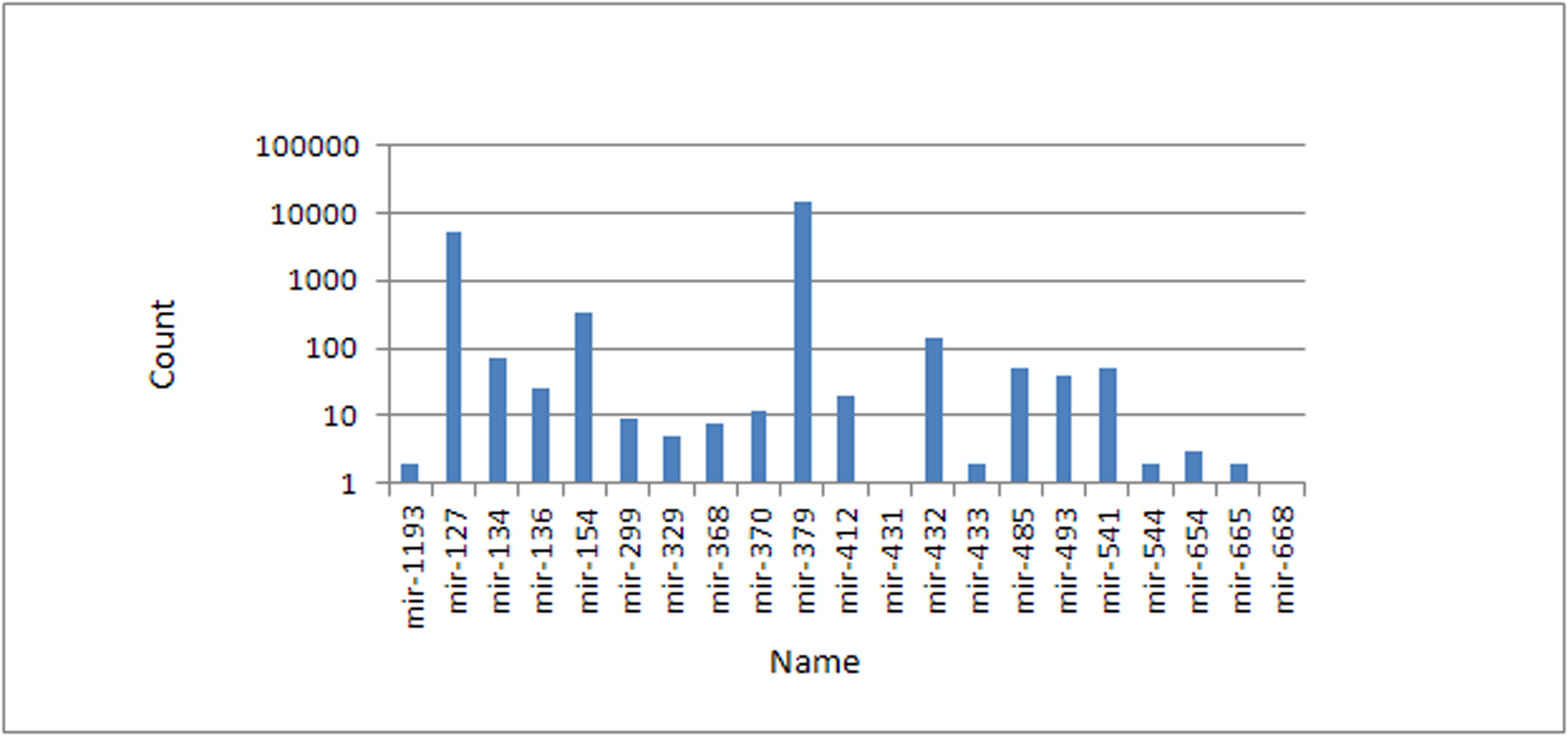

Supplement: Figure S2 — The abundance of the conserved miRNAs in goat. Most of the miRNAs were sequenced only a few times, whereas miR-127, miR-154 and miR-375 were sequenced thousands of times. Goat contains a large and diverse sRNA population at the hair growth stage. (TIF) [file pone.0050001.s002.tif]
