# Supplementary material for: Identification of Conserved and Novel microRNAs in Cashmere Goat Skin by Deep Sequencing
Source: PLoS One. 2012 Dec 7;7(12):e50001. doi: 10.1371/journal.pone.0050001 (PMC3517574; doi:10.1371/journal.pone.0050001)
Supplement: Table S1 — Summary of read signatures that match various RNAs. (DOC) [file pone.0050001.s003.doc]

Table S1, Summary of read signatures that match various RNAs

| Locus class | Unique sRNAs | Percent (%) | Total sRNAs | Percent (%) |
| --- | --- | --- | --- | --- |
| Small RNA matching protein-coding genes（77.72%）（85.47%） | | | | |
| exon_antisense | 164 | 0.03% | 178 | 0.00% |
| exon_sense | 19838 | 4.13% | 21076 | 0.20% |
| intron_antisense | 712 | 0.15% | 900 | 0.01% |
| intron_sense | 4469 | 0.93% | 11506 | 0.11% |
| miRNA | 3381 | 0.70% | 6113553 | 57.43% |
| Other smallRNAs | 345058 | 71.78% | 2948055 | 27.70% |
| Nonprotein-coding RNAs（22.28%）（14.53%） | | | | |
| rRNA | 66770 | 13.89% | 768202 | 7.22% |
| tRNA | 17615 | 3.66% | 648147 | 6.09% |
| scRNA | 226 | 0.05% | 1235 | 0.01% |
| snRNA | 2798 | 0.58% | 16866 | 0.16% |
| snoRNA | 5196 | 1.08% | 91607 | 0.86% |
| srpRNA | 1298 | 0.27% | 5822 | 0.05% |
| repeat | 13218 | 2.75% | 17320 | 0.16% |
| Total | 480743 | 100% | 10644467 | 100% |
