# Supplementary material for: Identification of Conserved and Novel microRNAs in Cashmere Goat Skin by Deep Sequencing
Source: PLoS One. 2012 Dec 7;7(12):e50001. doi: 10.1371/journal.pone.0050001 (PMC3517574; doi:10.1371/journal.pone.0050001)
Supplement: Table S2 — Conserved miRNA family analysis. “−” indicates no expression, “+” indicates expression. (DOC) [file pone.0050001.s004.doc]

Table S2, conserved miRNA family analysis

| Name | Sus | Equus | Ovis | Bos | Capra | Count | Number | Family members |
| --- | --- | --- | --- | --- | --- | --- | --- | --- |
| mir-1193 | - | - | + | + | + | 2 | 1 | 1193 |
| mir-127 | + | + | + | + | + | 5235 | 1 | 127 |
| mir-134 | - | + | + | + | + | 74 | 1 | 134 |
| mir-136 | + | + | + | + | + | 25 | 1 | 136 |
| mir-154 | + | + | + | + | + | 342 | 18 | 154a, 154b,323a, 323b, 323c,381, 382, 1185, 369, 377, 409, 410, 487a, 487b, 494, 655, 539,496 |
| mir-299 | + | + | + | + | + | 9 | 1 | 299 |
| mir-329 | - | + | + | + | + | 5 | 4 | 329a,329b,495, 543 |
| mir-368 | - | - | + | - | + | 8 | 5 | 376a, 376b,376c, 376d,376e |
| mir-370 | - | + | + | + | + | 12 | 1 | 370 |
| mir-379 | + | + | + | + | + | 14461 | 6 | 1197,379, 380,758, 411a,411b |
| mir-412 | - | + | + | + | + | 20 | 1 | 412 |
| mir-431 | - | + | + | + | + | 1 | 1 | 431 |
| mir-432 | + | + | + | + | + | 146 | 1 | 432 |
| mir-433 | - | + | + | + | + | 2 | 1 | 433 |
| mir-485 | - | + | + | + | + | 51 | 1 | 485 |
| mir-493 | - | + | + | + | + | 41 | 1 | 493 |
| mir-541 | - | + | + | + | + | 49 | 1 | 541 |
| mir-544 | - | + | + | + | + | 2 | 1 | 544 |
| mir-654 | - | - | + | + | + | 3 | 1 | 654 |
| mir-665 | - | - | + | + | + | 2 | 1 | 665 |
| mir-668 | - | - | + | - | + | 1 | 1 | 668 |
