# Supplementary material for: Identification of Conserved and Novel microRNAs in Cashmere Goat Skin by Deep Sequencing
Source: PLoS One. 2012 Dec 7;7(12):e50001. doi: 10.1371/journal.pone.0050001 (PMC3517574; doi:10.1371/journal.pone.0050001)
Supplement: Table S3 — Novel miRNAs analysis. As shown in table S3, we attained the result by comparing with the bovine genome information. mfe is the minimum folding free energy index. (DOC) [file pone.0050001.s005.doc]

Table S3, novel miRNAs analysis

| No. | Name | Count | location | Length(nt) | mfe(kcal/mol) | Pre-Sequence | miRNA Sequence |
| --- | --- | --- | --- | --- | --- | --- | --- |
| 1 | chi-mir-m1 | 7 | chr10:21244990:21245062:+ | 73 | -34.54 | GATTGGTGCTCCCAGGGATGTAGCTCCTAGTGCTGACCCTCCCTCTGGGGGCCCATCCCTGCAGTGCCTTTCT | CCCAGGGATGTAGCTCCTAGTGC |
| 2 | chi-mir-m2 | 9 | chr10:66857950:66858041:+ | 92 | -31.8 | TAGTGGTTAGTACTCTGCGTTGTGGCCGCAGCAACCTCGGTTCGAATCCGAGTCACGGCATTGTGGCAACAATGGCACGGCAAGGGACCTCT | TTGTGGCAACAATGGCACGGCA |
| 3 | chi-mir-m3 | 72 | chr11:89654171:89654250:- | 80 | -33.49 | GTTGTAGGGATACAGTGACCAGGTGACGACGGATTTCTCAAGTAACAACCTCATGGCTTGGTCACAGTGTCCATATAACA | TACAGTGACCAGGTGACGACGG |
| 4 | chi-mir-m4 | 22 | chr13:58300859:58300945:+ | 87 | -42.4 | CTGAGCCTGCTGCCAAGCCCACGTTCAAAGGCTGTTTCTTCAAAGTTAATGGTGCCGCCTTTGAGCTGGGAAGGGAAGCGGGCAGTT | TGCCAAGCCCACGTTCAAAGG |
| 5 | chi-mir-m5 | 23 | chr14:29053702:29053781:- | 80 | -32.6 | GGCTCCGCTCTTGGCATTCACCGCGTGCCTTAATTGTATGACATTAAATCAAGGTCCGCTGTGAACACGGAGGGCAGAGT | TCAAGGTCCGCTGTGAACACGG |
| 6 | chi-mir-m6 | 13 | chr16:29014961:29015037:- | 77 | -41.1 | GGGTTGGCCAATAAGTTCATTCGGAGTTGTCCACGCGATCATCTGGAAAACTCTGTATGAACTTTTTGGTCAACCTG | ATAAGTTCATTCGGAGTTGTC |
| 7 | chi-mir-m7 | 451 | chr18:35987042:35987127:+ | 86 | -47.1 | TGTGTCCTGCCAGTGGTTTTACCCTATGGTAGGTTACGTCATGCTGTTCTACCACAGGGTAGAACCACGGACAGGATACCGGGGCA | CAGTGGTTTTACCCTATGGTAG |
| 8 | chi-mir-m8 | 32 | chr18:3419503:3419583:- | 81 | -42.9 | GTCCTGGCTTTCAGAGCTGTGGTTCAAAAGCTGTTGGGATCACTTAGAAGACTTTTGAACCACAAATCTGAAAACCAGGGA | TCAGAGCTGTGGTTCAAAAGC |
| 9 | chi-mir-m9 | 6 | chr19:57405987:57406063:+ | 77 | -33.57 | GCGTGTCCACTTGGAACGCAGGCTGTCAGCACTGCAGACGTCCTGATGGCCTGTGCTTCCCCCTTCAGCTTCACGCG | TTGGAACGCAGGCTGTCAGCAC |
| 10 | chi-mir-m10 | 909 | chr1:65888804:65888882:- | 79 | -31.3 | CTGTTCTCTTGAGAGATCAGAGGCGCAGAGTGCGTCAATGTCAATGAAGCCTGTGCCTTTTACCTCTTTAAGAGCGCAC | GAGAGATCAGAGGCGCAGAGT |
| 11 | chi-mir-m11 | 33 | chr21:66014117:66014196:+ | 80 | -35.2 | AGATGTTCGAAAGGAGGTTGTCCGTGATGTATTTGCTTTATTTGTGGCAGATATTGCACGGTTGATCTCTTTTCTTCATC | AGATATTGCACGGTTGATCTCT |
| 12 | chi-mir-m12 | 43 | chr21:66028347:66028420:+ | 74 | -34.9 | GGGAGTGGATGGTTGATCAGAGAACATACATTTTGTCAATGATGTATGTCAACTGATCCACAGTCCCTCCCTAT | GGTTGATCAGAGAACATACATT |
| 13 | chi-mir-m13 | 10 | chr21:66037226:66037305:+ | 80 | -33.9 | GTACTTGAAGAGAGGTCTTCCATGGTGCATTCGCTTTATTCTTTGACGAATCATACATGGTTGACCTTTTTTTAGGTATC | AGAGGTCTTCCATGGTGCATTCG |
| 14 | chi-mir-m14 | 8 | chr23:3760912:3760994:+ | 83 | -52.2 | CTTGTGGGACAGATCTGTCCTGAAACCAGCATAAAGTAGCAGCTGCCATACTGGTTTCAGGACAGATCTCTCCCACAAGCTGG | AGATCTGTCCTGAAACCAGCA |
| 15 | chi-mir-m15 | 8 | chr23:13431292:13431382:+ | 91 | -40.2 | TGGAGCCCAGCTGGAAATGTTCTAGCCAAAAAAGTTTGCCAAGAACCACTGTGTCTTTTTTTTTTGCTGGAACATTTCTGGTTGTGCTTCT | TTTTTTGCTGGAACATTTCTGG |
| 16 | chi-mir-m16 | 99 | chr26:34058289:34058367:+ | 79 | -37 | GGGAGGGGCGTAGAGAAGCACTGGGGGAAAGTCTTAGAAGTAAGATGCTGCCCCTTGATGCTTGTCTTTATCCCTTTCA | TAGAGAAGCACTGGGGGAAAGT |
| 17 | chi-mir-m17 | 5 | chr26:24439300:24439375:- | 76 | -30.74 | CCTACCAATCTCGACCGGACCTCGACCGGCTCGTCTATATTGCCAATCGACTCGGCGTGGCGTCGGTCGTGGTAGA | TCGACCGGACCTCGACCGGCTCG |
| 18 | chi-mir-m18 | 10 | chr5:61789475:61789550:- | 76 | -25.12 | TGAGCAAGAGTAAGGAAAAGGCTTGTTAGGAGACCTCAGGCTCCTCCTAACTCAGCCCTTCCTTTCCAGTGGCAGA | TAAGGAAAAGGCTTGTTAGG |
| 19 | chi-mir-m19 | 7 | chr7:16299260:16299332:- | 73 | -33.3 | GCCCTGGGGTTAGGGGGAGCGGTTGGAGGACTGGGGGCTCAGCCTTCCTACCTCTCTCCGTAGGAGCCTGGTG | TAGGGGGAGCGGTTGGAGGACT |
| 20 | chi-mir-m20 | 23 | chr8:9359007:9359088:- | 82 | -27.6 | AGCCCCATTCTTGGCATTCACCGCGTGCCTTAATTGTATGGACATTTAAATCAAGGTCCGCTGTGAACACGGAGAGAGAGGC | TCAAGGTCCGCTGTGAACACGG |
| 21 | chi-mir-m21 | 225 | chrUn.004.245:10867:10949:+ | 83 | -24.72 | AGTCATTTTGAGAGGTAAAAAATTGATTTGACTAGTTCTTTAACACATCTAGCAAATCATTTTTTACTCTCCAAAAAGAACTC | AGAGGTAAAAAATTGATTTGACT |
| 22 | chi-mir-m22 | 1408 | chrX:21829425:21829509:- | 85 | -32.8 | ATGAATGCGAAAACCTCAGTCAGCCTTGTGGATGTATGTTCTGCAGACCTGACATCTAGAGGACTGACTGAAATTTTCACTTTCA | ATCTAGAGGACTGACTGAAATT |
