# Supplementary material for: Identification of Conserved and Novel microRNAs in Cashmere Goat Skin by Deep Sequencing
Source: PLoS One. 2012 Dec 7;7(12):e50001. doi: 10.1371/journal.pone.0050001 (PMC3517574; doi:10.1371/journal.pone.0050001)
Supplement: Information S1 — 68 conserved miRNAs in goat. Description of data: These miRNAs are conserved in goat and have been reported in NCBI. (DOC) [file pone.0050001.s006.doc]

Supporting information S1: 68 conserved miRNAs in goat

| Name | Count | Name | Count | Name | Count |
| --- | --- | --- | --- | --- | --- |
| miR-143 | 1344689 | miR-23b-3p | 12525 | miR-125a | 1267 |
| let-7a | 1255397 | miR-93 | 12487 | miR-30c | 1267 |
| let-7b | 1193996 | miR-23a | 10730 | miR-106b | 1140 |
| let-7f | 398709 | let-7i | 8681 | miR-31 | 1060 |
| let-7c | 299649 | miR-99b | 8214 | miR-30b-5p | 845 |
| miR-21 | 160762 | miR-320 | 8152 | miR-15a | 558 |
| miR-200c | 76101 | miR-191 | 6432 | miR-221 | 718 |
| miR-30d | 73310 | miR-205 | 5963 | miR-193a-5p | 531 |
| miR-30a-5p | 68298 | miR-125b | 5740 | miR-19b | 489 |
| miR-10b | 57917 | miR-26b | 5202 | miR-199b | 420 |
| miR-103 | 44327 | miR-100 | 4995 | miR-342 | 258 |
| miR-26a | 42483 | miR-25 | 4060 | miR-15b | 254 |
| miR-27b | 39937 | let-7d | 4005 | miR-210 | 138 |
| let-7g | 38013 | miR-200b | 3562 | miR-107 | 132 |
| miR-30e-5p | 28485 | miR-141 | 3536 | miR-339 | 130 |
| miR-126 | 21246 | miR-130a | 3230 | miR-19a | 81 |
| let-7e | 19659 | miR-92 | 3221 | miR-335 | 64 |
| miR-140 | 15583 | miR-20a | 2883 | miR-24 | 21 |
| miR-27a-3p | 14101 | miR-200a | 2419 | miR-106 | 20 |
| miR-181a | 14076 | miR-99a | 2244 | miR-204 | 11 |
| miR-152 | 13760 | miR-98 | 2051 | miR-195 | 4 |
| miR-10a | 13225 | miR-16a | 2019 | miR-20b | 2 |
| miR-199a-3p | 12895 | miR-17-5p | 1868 |  |  |
