# Supplementary material for: Identification of Conserved and Novel microRNAs in Cashmere Goat Skin by Deep Sequencing
Source: PLoS One. 2012 Dec 7;7(12):e50001. doi: 10.1371/journal.pone.0050001 (PMC3517574; doi:10.1371/journal.pone.0050001)
Supplement: Information S2 — Novel miRNAs identified in the present study. Description of data: 248 miRNA sequences were conserved in other species and have not been reported in goat. (DOC) [file pone.0050001.s007.doc]

Supporting information S2: Novel miRNAs identified in the present study.

| Name | Count | Name | Count | Name | Count |
| --- | --- | --- | --- | --- | --- |
| miR-378 | 155553 | miR-193b | 778 | miR-2478 | 122 |
| miR-148a | 125764 | miR-34c | 763 | miR-122 | 121 |
| miR-24-3p | 76282 | miR-744 | 697 | miR-215 | 118 |
| miR-146b | 62327 | miR-27a-5p | 662 | miR-150 | 114 |
| miR-182 | 38075 | miR-185 | 643 | miR-96 | 111 |
| miR-184 | 37292 | miR-142* | 635 | miR-362-5p | 105 |
| miR-101 | 26457 | miR-375 | 571 | miR-338 | 102 |
| miR-151 | 24115 | miR-206 | 568 | miR-197 | 101 |
| miR-423-5p | 14262 | miR-429 | 549 | miR-1271 | 98 |
| miR-379 | 13210 | miR-451 | 533 | miR-455 | 91 |
| miR-148b | 11854 | miR-486 | 496 | miR-455* | 90 |
| miR-183 | 11520 | miR-424 | 481 | miR-139 | 86 |
| miR-1 | 8442 | miR-374b | 469 | miR-211 | 86 |
| miR-127 | 5235 | miR-214 | 441 | miR-18a | 84 |
| miR-29a | 4634 | miR-374a | 415 | miR-129 | 80 |
| miR-192 | 4260 | miR-3431 | 333 | miR-129-5p | 80 |
| miR-155 | 4113 | miR-1468 | 300 | miR-877 | 73 |
| miR-92a | 3471 | miR-296 | 294 | miR-301a | 72 |
| miR-452 | 3132 | miR-224 | 258 | miR-193a-3p | 70 |
| miR-196a | 2409 | miR-142 | 257 | miR-2887 | 69 |
| miR-186 | 2312 | miR-222 | 238 | miR-450 | 65 |
| miR-1839 | 2137 | miR-361 | 237 | miR-365-3p | 61 |
| miR-151* | 2118 | miR-382 | 222 | miR-196b | 60 |
| miR-16b | 2034 | miR-199c | 215 | miR-421 | 56 |
| miR-126* | 1951 | miR-194 | 213 | miR-484 | 56 |
| miR-181b | 1823 | miR-2411 | 211 | miR-345-3p | 55 |
| miR-130b | 1575 | miR-497 | 200 | miR-1940 | 53 |
| miR-28 | 1484 | miR-365-5p | 193 | miR-328 | 52 |
| miR-532 | 1465 | miR-29c | 183 | miR-485 | 51 |
| miR-146a | 1434 | miR-1307 | 173 | miR-425-5p | 50 |
| miR-708 | 1299 | miR-218 | 166 | miR-330 | 47 |
| miR-411 | 1214 | miR-3432 | 146 | miR-219-3p | 45 |
| miR-2284x | 1205 | miR-432 | 146 | miR-181c | 44 |
| miR-7 | 1188 | miR-29b | 138 | miR-500 | 43 |
| miR-199a-5p | 962 | miR-17-3p | 137 | miR-2318 | 42 |
| miR-22-3p | 951 | miR-874 | 134 | miR-381 | 42 |
| miR-423-3p | 854 | miR-217 | 128 | miR-133a | 41 |
| miR-9 | 831 | miR-181d | 126 | miR-493 | 41 |
| miR-660 | 807 | miR-128 | 123 | miR-34a | 40 |
| miR-145 | 797 | miR-30f | 123 | miR-499 | 38 |
| Name | Count | Name | Count | Name | Count |
| miR-92b | 37 | miR-21* | 12 | miR-543 | 2 |
| miR-2355 | 37 | miR-370 | 12 | miR-433 | 2 |
| miR-190a | 36 | miR-502b | 12 | miR-153 | 2 |
| miR-95 | 33 | miR-216a | 12 | miR-23b | 2 |
| miR-22-5p | 33 | miR-655 | 11 | miR-876 | 2 |
| miR-216b | 33 | miR-30b-3p | 11 | miR-2424 | 2 |
| miR-769 | 32 | miR-2474 | 11 | miR-592 | 2 |
| miR-340 | 30 | miR-502a | 10 | miR-677 | 2 |
| miR-628 | 29 | miR-324 | 9 | miR-665 | 2 |
| miR-138 | 29 | miR-132 | 9 | miR-29d | 2 |
| miR-2483 | 28 | miR-2483* | 9 | miR-212 | 2 |
| miR-542-5p | 28 | miR-2285b | 9 | miR-2330 | 2 |
| miR-1298 | 27 | miR-299 | 9 | miR-2411* | 2 |
| miR-369-3p | 26 | miR-124a | 8 | miR-488 | 2 |
| miR-1343* | 25 | miR-124b | 8 | miR-1185 | 2 |
| miR-135a | 25 | miR-2387 | 8 | miR-2290 | 2 |
| miR-136 | 25 | miR-301b | 8 | miR-331 | 1 |
| miR-99a* | 24 | miR-326 | 7 | miR-2307 | 1 |
| miR-1388-5p | 23 | miR-2366 | 7 | miR-551b | 1 |
| miR-380-3p | 23 | miR-2448 | 6 | miR-487a | 1 |
| miR-137 | 22 | miR-487b | 6 | miR-2331* | 1 |
| miR-885 | 22 | miR-133b | 6 | miR-2487 | 1 |
| miR-135b | 22 | miR-1814c | 5 | miR-1343 | 1 |
| miR-504 | 21 | miR-2310 | 5 | miR-545 | 1 |
| miR-767 | 20 | miR-410 | 5 | miR-329b | 1 |
| bta-let-7a* | 20 | miR-873 | 5 | miR-376d | 1 |
| miR-33a | 20 | miR-409 | 5 | miR-1197 | 1 |
| miR-491 | 20 | miR-1224 | 4 | miR-144 | 1 |
| miR-9* | 19 | miR-1434 | 4 | miR-582 | 1 |
| miR-187 | 19 | miR-345-5p | 4 | miR-1248 | 1 |
| miR-424* | 17 | miR-202 | 4 | miR-190b | 1 |
| miR-671 | 16 | miR-363 | 4 | miR-2285c | 1 |
| miR-1306 | 16 | miR-369-5p | 4 | miR-2355* | 1 |
| miR-425-3p | 15 | miR-362-3p | 4 | miR-2427 | 1 |
| miR-1296 | 15 | miR-129-3p | 4 | miR-2428 | 1 |
| miR-664 | 15 | miR-376b | 4 | miR-2447 | 1 |
| miR-494 | 14 | miR-339b | 3 | miR-2476 | 1 |
| miR-105a | 13 | miR-323 | 3 | miR-2904 | 1 |
| miR-1249 | 13 | miR-376c | 3 | miR-3604 | 1 |
| miR-380-5p | 13 | miR-448 | 3 | miR-545* | 1 |
| miR-505 | 13 | miR-188 | 2 | miR-656 | 1 |
| miR-454 | 12 | miR-495 | 2 | miR-760 | 1 |
| miR-223 | 12 | miR-490 | 2 |  |  |
