# Supplementary material for: Identification of Conserved and Novel microRNAs in Cashmere Goat Skin by Deep Sequencing
Source: PLoS One. 2012 Dec 7;7(12):e50001. doi: 10.1371/journal.pone.0050001 (PMC3517574; doi:10.1371/journal.pone.0050001)

Supporting information S3:Secondary structures of novel miRNAs in goat


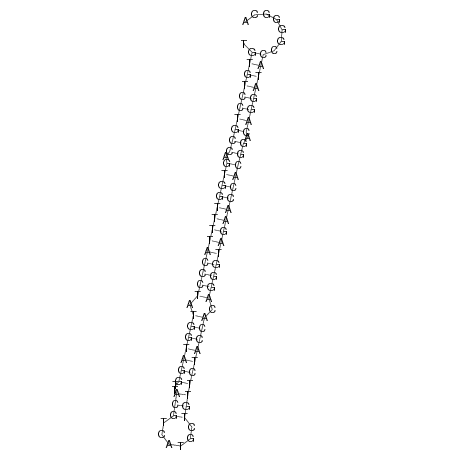
SY-1-m0001

SY-1-m0002


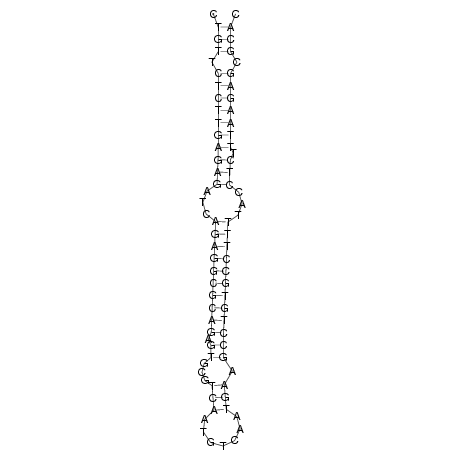


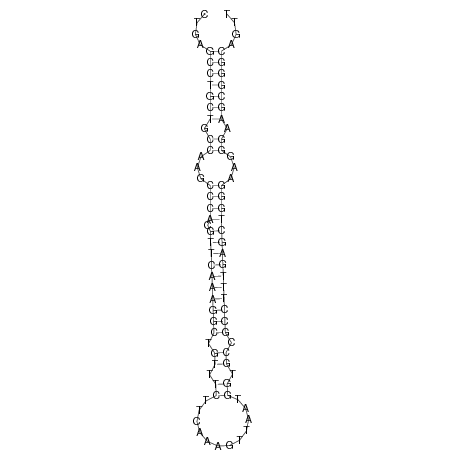
SY-1-m0003

SY-1-m0004


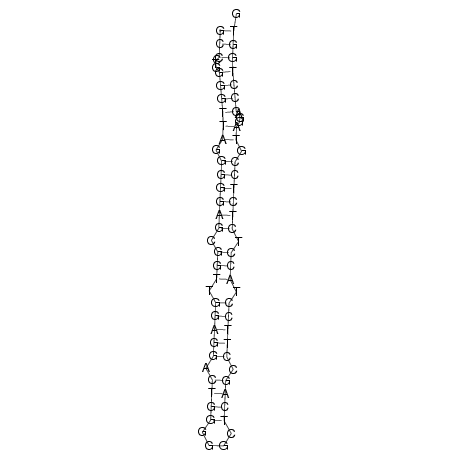
 SY-1-m0005


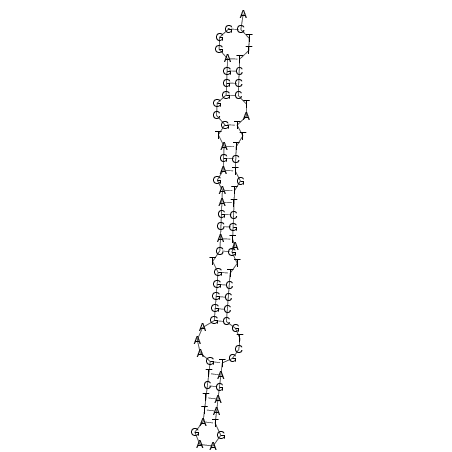


SY-1-m0006


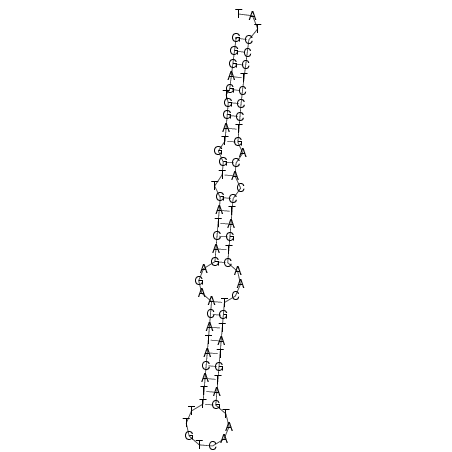


SY-1-m0007


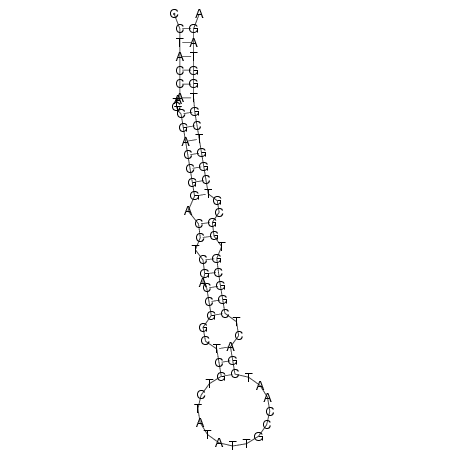


SY-1-m0008


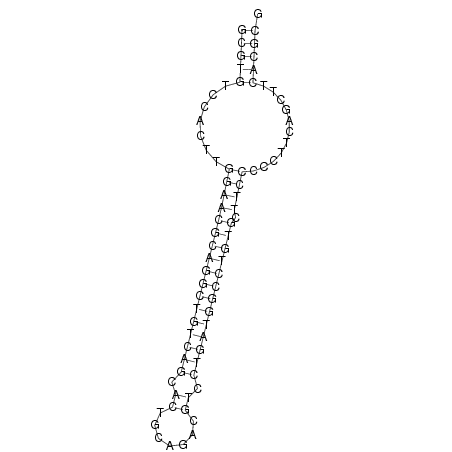


SY-1-m0009


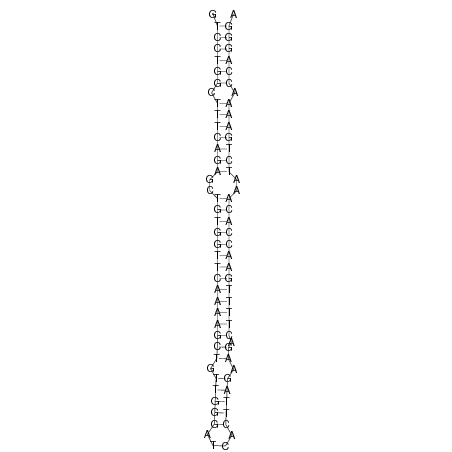


SY-1-m00010


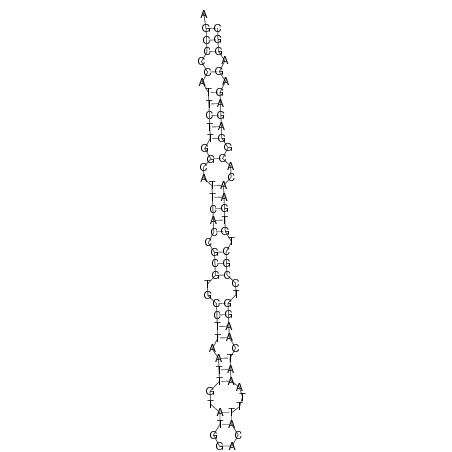


SY-1-m00011


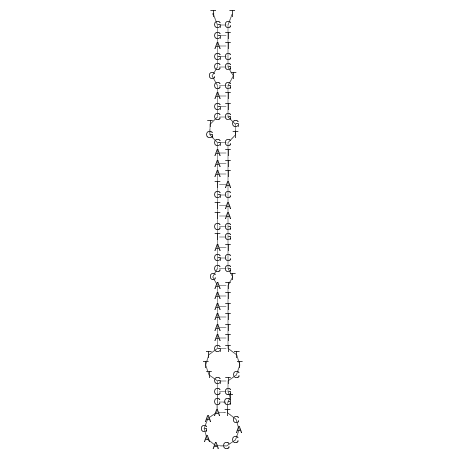


SY-1-m00012


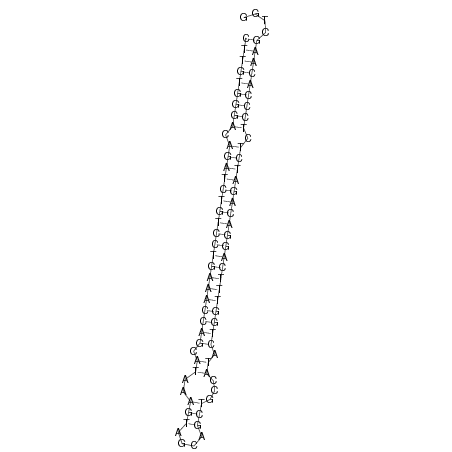


SY-1-m00013


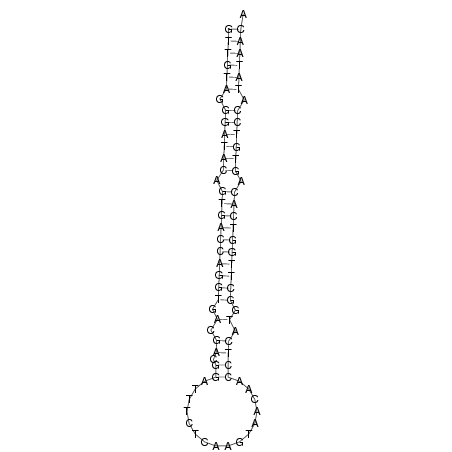


SY-1-m00014


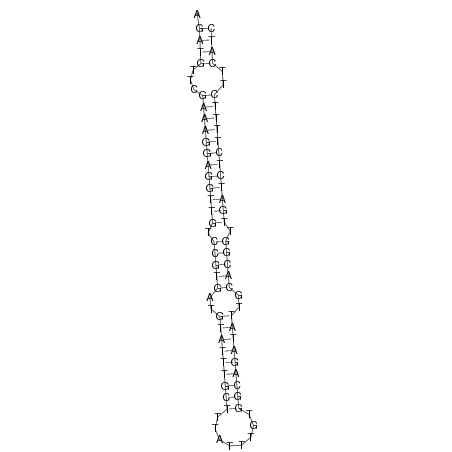


SY-1-m00015


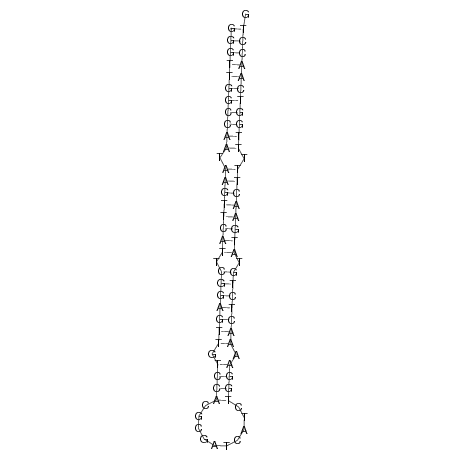


SY-1-m00016


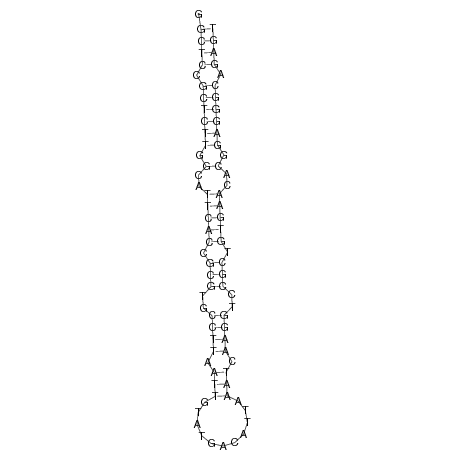


SY-1-m00017


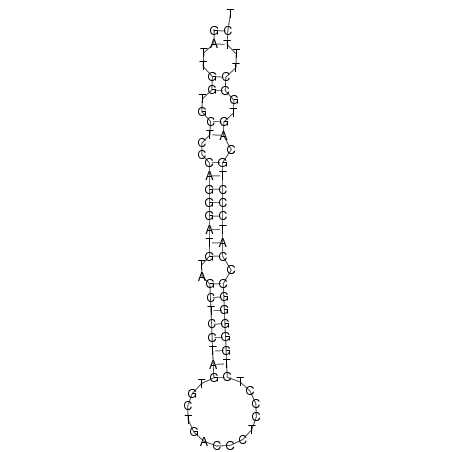


SY-1-m00018


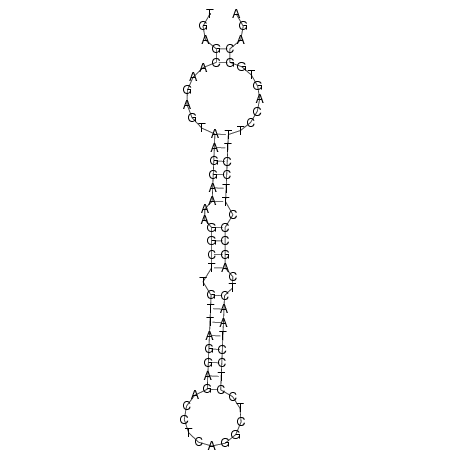


SY-1-m00019


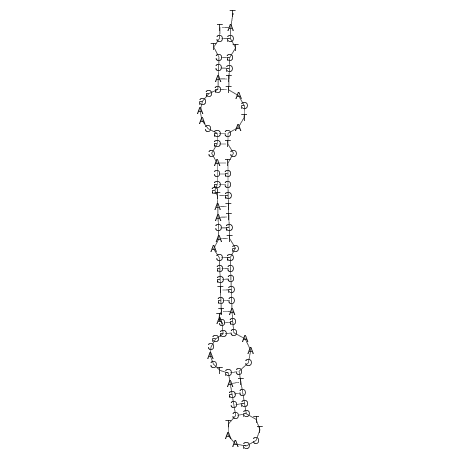


SY-1-m00020


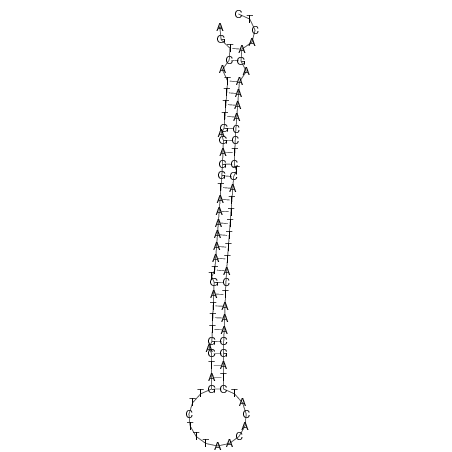


SY-1-m00021


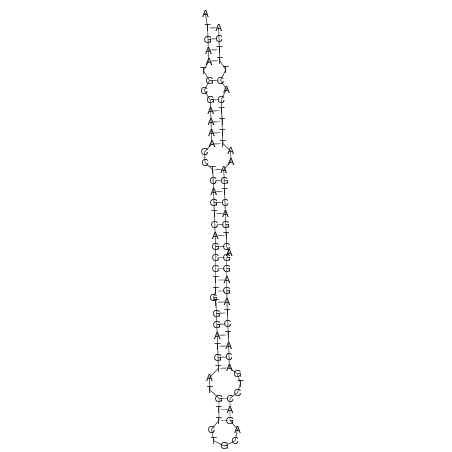


SY-1-m00022


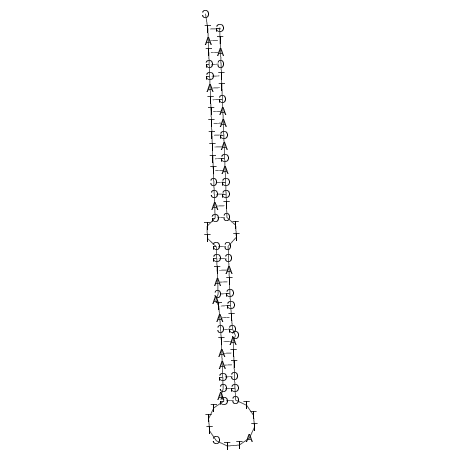

Supplement: Information S3 — Secondary structures of the precursors of the novel goat miRNAs. Description of data: This file shows detailed information of secondary structures of the precursors of the novel goat miRNAs. (DOC) [file pone.0050001.s008.doc]
