# Supplementary material for: Identification of Conserved and Novel microRNAs in Cashmere Goat Skin by Deep Sequencing
Source: PLoS One. 2012 Dec 7;7(12):e50001. doi: 10.1371/journal.pone.0050001 (PMC3517574; doi:10.1371/journal.pone.0050001)
Supplement: Information S4 — Predicted targets for the novel goat miRNAs. Description of data: This file shows detailed information of each miRNA hairpin. (DOC) [file pone.0050001.s009.doc]

Supporting information S4： detailed information of each miRNA hairpin.

SY-1-m0001 chr10:21244990:21245062:+ 73(nt) -34.54(kcal/mol)

GATTGGTGCTCCCAGGGATGTAGCTCCTAGTGCTGACCCTCCCTCTGGGGGCCCATCCCTGCAGTGCCTTTCT SY-1-m0001 7

((..((..((..((((((((..((((((((..............)))))))).)))))))).))..))..)).

**********CCCAGGGATGTAGCTCCTAGTGC**************************************** SY-1-m0001-5p 7

---------TCCCAGGGATGTAGCTCCTAGT------------------------------------------ t0233330 1

----------CCCAGGGATGTAGCTCCTAGTGC---------------------------------------- t0028265 6

SY-1-m0002 chr10:66857950:66858041:+ 92(nt) -31.80(kcal/mol)

TAGTGGTTAGTACTCTGCGTTGTGGCCGCAGCAACCTCGGTTCGAATCCGAGTCACGGCATTGTGGCAACAATGGCACGGCAAGGGACCTCT SY-1-m0002 9

.((.((((....((.((((((((.((((((((...(((((.......))))).....))..)))))).))))).))).)).....)))).))

************************************************************TTGTGGCAACAATGGCACGGCA********** SY-1-m0002-3p 9

------------------------------------------------------------TTGTGGCAACAATGGCACGGC----------- t0076002 2

------------------------------------------------------------TTGTGGCAACAATGGCACGGCA---------- t0032373 5

-------------------------------------------------------------TGTGGCAACAATGGCACGGCA---------- t0067644 2

SY-1-m0003 chr11:89654171:89654250:- 80(nt) -33.49(kcal/mol)

GTTGTAGGGATACAGTGACCAGGTGACGACGGATTTCTCAAGTAACAACCTCATGGCTTGGTCACAGTGTCCATATAACA SY-1-m0003 72

((((((.((((((.((((((((((.(.((.((................)))).).)))))))))).)))))).)))))).

**********TACAGTGACCAGGTGACGACGG************************************************ SY-1-m0003-5p 72

----------TACAGTGACCAGGTGACGACG------------------------------------------------- t0025896 7

----------TACAGTGACCAGGTGACGACGG------------------------------------------------ t0005929 50

----------TACAGTGACCAGGTGACGACGGA----------------------------------------------- t0014800 14

----------TACAGTGACCAGGTGACGACGGAT---------------------------------------------- t0268958 1

SY-1-m0004 chr13:58300859:58300945:+ 87(nt) -42.40(kcal/mol)

CTGAGCCTGCTGCCAAGCCCACGTTCAAAGGCTGTTTCTTCAAAGTTAATGGTGCCGCCTTTGAGCTGGGAAGGGAAGCGGGCAGTT SY-1-m0004 22

....(((((((.((...((((.((((((((((.((.((............)).)).))))))))))))))...)).)))))))....

**********TGCCAAGCCCACGTTCAAAGG******************************************************** SY-1-m0004-5p 22

----------TGCCAAGCCCACGTTCAAAGG-------------------------------------------------------- t0014300 15

----------TGCCAAGCCCACGTTCAAAGGC------------------------------------------------------- t0026155 6

----------TGCCAAGCCCACGTTCAAAGGCT------------------------------------------------------ t0193232 1

SY-1-m0005 chr14:29053702:29053781:- 80(nt) -32.60(kcal/mol)

GGCTCCGCTCTTGGCATTCACCGCGTGCCTTAATTGTATGACATTAAATCAAGGTCCGCTGTGAACACGGAGGGCAGAGT SY-1-m0005 23

.((((.((((((.(..(((((.(((..((((.(((...........))).))))..))).)))))..).)))))).))))

************************************************TCAAGGTCCGCTGTGAACACGG********** SY-1-m0005-3p 23

------------------------------------------------TCAAGGTCCGCTGTGAACACG----------- t0263485 1

------------------------------------------------TCAAGGTCCGCTGTGAACACGG---------- t0012421 18

-------------------------------------------------CAAGGTCCGCTGTGAACACGG---------- t0078957 2

-------------------------------------------------CAAGGTCCGCTGTGAACACGGA--------- t0083293 2

SY-1-m0006 chr16:29014961:29015037:- 77(nt) -41.10(kcal/mol)

GGGTTGGCCAATAAGTTCATTCGGAGTTGTCCACGCGATCATCTGGAAAACTCTGTATGAACTTTTTGGTCAACCTG SY-1-m0006 13

(((((((((((.((((((((.(((((((.((((..........)))).))))))).)))))))).))))))))))).

**********ATAAGTTCATTCGGAGTTGTC********************************************** SY-1-m0006-5p 13

----------ATAAGTTCATTCGGAGTTGT----------------------------------------------- t0039102 4

----------ATAAGTTCATTCGGAGTTGTC---------------------------------------------- t0037907 4

----------ATAAGTTCATTCGGAGTTGTCC--------------------------------------------- t0041085 3

-----------TAAGTTCATTCGGAGTTGTC---------------------------------------------- t0153212 1

-----------TAAGTTCATTCGGAGTTGTCCA-------------------------------------------- t0376219 1

SY-1-m0007 chr18:35987042:35987127:+ 86(nt) -47.10(kcal/mol)

TGTGTCCTGCCAGTGGTTTTACCCTATGGTAGGTTACGTCATGCTGTTCTACCACAGGGTAGAACCACGGACAGGATACCGGGGCA SY-1-m0007 451

.((((((((((.(((((((((((((.(((((((..(((......)))))))))).))))))))))))))).)))))))).......

**********CAGTGGTTTTACCCTATGGTAG****************************************************** SY-1-m0007-5p 445

******************************************************CAGGGTAGAACCACGGACAGGA********** SY-1-m0007-3p 6

----------CAGTGGTTTTACCCTATGG--------------------------------------------------------- t0395787 1

----------CAGTGGTTTTACCCTATGGTA------------------------------------------------------- t0050535 3

----------CAGTGGTTTTACCCTATGGTAG------------------------------------------------------ t0001023 435

----------CAGTGGTTTTACCCTATGGTAGG----------------------------------------------------- t0041243 3

-----------AGTGGTTTTACCCTATGGTAG------------------------------------------------------ t0042490 3

------------------------------------------------------CAGGGTAGAACCACGGACAGG----------- t0291915 1

------------------------------------------------------CAGGGTAGAACCACGGACAGGA---------- t0031426 5

SY-1-m0008 chr18:3419503:3419583:- 81(nt) -42.90(kcal/mol)

GTCCTGGCTTTCAGAGCTGTGGTTCAAAAGCTGTTGGGATCACTTAGAAGACTTTTGAACCACAAATCTGAAAACCAGGGA SY-1-m0008 32

.((((((.(((((((..(((((((((((((((.(((((....))))).)).)))))))))))))..))))))).)))))).

**********TCAGAGCTGTGGTTCAAAAGC************************************************** SY-1-m0008-5p 32

----------TCAGAGCTGTGGTTCAAAAGC-------------------------------------------------- t0009191 28

----------TCAGAGCTGTGGTTCAAAAGCT------------------------------------------------- t0040383 4

SY-1-m0009 chr19:57405987:57406063:+ 77(nt) -33.57(kcal/mol)

GCGTGTCCACTTGGAACGCAGGCTGTCAGCACTGCAGACGTCCTGATGGCCTGTGCTTCCCCCTTCAGCTTCACGCG SY-1-m0009 6

(((((.......(((((((((((((((((.((.......)).))))))))))))).))))...........))))).

**********TTGGAACGCAGGCTGTCAGCAC********************************************* SY-1-m0009-5p 6

----------TTGGAACGCAGGCTGTCAG------------------------------------------------ t0447647 1

----------TTGGAACGCAGGCTGTCAGC----------------------------------------------- t0403505 1

----------TTGGAACGCAGGCTGTCAGCA---------------------------------------------- t0425699 1

----------TTGGAACGCAGGCTGTCAGCAC--------------------------------------------- t0047824 3

SY-1-m0010 chr1:65888804:65888882:- 79(nt) -31.30(kcal/mol)

CTGTTCTCTTGAGAGATCAGAGGCGCAGAGTGCGTCAATGTCAATGAAGCCTGTGCCTTTTACCTCTTTAAGAGCGCAC SY-1-m0010 909

.(((.((((((((((...((((((((((.((...(((.......))).))))))))))))...))).))))))).))).

**********GAGAGATCAGAGGCGCAGAGT************************************************ SY-1-m0010-5p 909

---------TGAGAGATCAGAGGCGCAGA-------------------------------------------------- t0467628 1

---------TGAGAGATCAGAGGCGCAGAGT------------------------------------------------ t0057493 2

----------GAGAGATCAGAGGCGCAG--------------------------------------------------- t0143000 1

----------GAGAGATCAGAGGCGCAGA-------------------------------------------------- t0016600 12

----------GAGAGATCAGAGGCGCAGAG------------------------------------------------- t0008720 30

----------GAGAGATCAGAGGCGCAGAGT------------------------------------------------ t0000552 858

----------GAGAGATCAGAGGCGCAGAGTGC---------------------------------------------- t0035511 4

-----------AGAGATCAGAGGCGCAGAGT------------------------------------------------ t0274890 1

SY-1-m0011 chr21:66014117:66014196:+ 80(nt) -35.20(kcal/mol)

AGATGTTCGAAAGGAGGTTGTCCGTGATGTATTTGCTTTATTTGTGGCAGATATTGCACGGTTGATCTCTTTTCTTCATC SY-1-m0011 33

.((((...((((((((((((.(((((..(((((((((........)))))))))..))))).))))))))))))..))))

************************************************AGATATTGCACGGTTGATCTCT********** SY-1-m0011-3p 33

------------------------------------------------AGATATTGCACGGTTGATCT------------ t0078262 2

------------------------------------------------AGATATTGCACGGTTGATCTC----------- t0057157 2

------------------------------------------------AGATATTGCACGGTTGATCTCT---------- t0008982 29

SY-1-m0012 chr21:66028347:66028420:+ 74(nt) -34.90(kcal/mol)

GGGAGTGGATGGTTGATCAGAGAACATACATTTTGTCAATGATGTATGTCAACTGATCCACAGTCCCTCCCTAT SY-1-m0012 43

(((((.((((.((.((((((...(((((((((........)))))))))...)))))).)).)))))))))...

**********GGTTGATCAGAGAACATACATT****************************************** SY-1-m0012-5p 30

******************************************TGTATGTCAACTGATCCACAGT********** SY-1-m0012-3p 13

--------ATGGTTGATCAGAGAACATAC--------------------------------------------- t0022686 8

--------ATGGTTGATCAGAGAACATACA-------------------------------------------- t0029219 6

----------GGTTGATCAGAGAACATA---------------------------------------------- t0283018 1

----------GGTTGATCAGAGAACATACA-------------------------------------------- t0377971 1

----------GGTTGATCAGAGAACATACAT------------------------------------------- t0040327 4

----------GGTTGATCAGAGAACATACATT------------------------------------------ t0020947 9

----------GGTTGATCAGAGAACATACATTT----------------------------------------- t0329336 1

------------------------------------------TGTATGTCAACTGATCCACA------------ t0060275 2

------------------------------------------TGTATGTCAACTGATCCACAG----------- t0075916 2

------------------------------------------TGTATGTCAACTGATCCACAGT---------- t0023431 8

--------------------------------------------TATGTCAACTGATCCACAG----------- t0334206 1

SY-1-m0013 chr21:66037226:66037305:+ 80(nt) -33.90(kcal/mol)

GTACTTGAAGAGAGGTCTTCCATGGTGCATTCGCTTTATTCTTTGACGAATCATACATGGTTGACCTTTTTTTAGGTATC SY-1-m0013 10

(((((((((((((((((..((((((((.((((((..........).)))))))).)))))..))))))))))))))))).

**********AGAGGTCTTCCATGGTGCATTCG*********************************************** SY-1-m0013-5p 10

----------AGAGGTCTTCCATGGTGCAT-------------------------------------------------- t0459469 1

----------AGAGGTCTTCCATGGTGCATTC------------------------------------------------ t0057082 2

----------AGAGGTCTTCCATGGTGCATTCG----------------------------------------------- t0030022 5

-----------GAGGTCTTCCATGGTGCATTCG----------------------------------------------- t0061494 2

SY-1-m0014 chr23:3760912:3760994:+ 83(nt) -52.20(kcal/mol)

CTTGTGGGACAGATCTGTCCTGAAACCAGCATAAAGTAGCAGCTGCCATACTGGTTTCAGGACAGATCTCTCCCACAAGCTGG SY-1-m0014 8

(((((((((.(((((((((((((((((((.((..(((....)))...)).))))))))))))))))))).)))))))))....

**********AGATCTGTCCTGAAACCAGCA**************************************************** SY-1-m0014-5p 5

**************************************************CTGGTTTCAGGACAGATCTCTCC********** SY-1-m0014-3p 3

----------AGATCTGTCCTGAAACCAGC----------------------------------------------------- t0084296 2

----------AGATCTGTCCTGAAACCAGCA---------------------------------------------------- t0070277 2

----------AGATCTGTCCTGAAACCAGCAT--------------------------------------------------- t0132473 1

--------------------------------------------------CTGGTTTCAGGACAGATCTCTCC---------- t0062455 2

---------------------------------------------------TGGTTTCAGGACAGATCTCTC----------- t0152426 1

SY-1-m0015 chr23:13431292:13431382:+ 91(nt) -40.20(kcal/mol)

TGGAGCCCAGCTGGAAATGTTCTAGCCAAAAAAGTTTGCCAAGAACCACTGTGTCTTTTTTTTTTGCTGGAACATTTCTGGTTGTGCTTCT SY-1-m0015 8

.(((((.((((..(((((((((((((.(((((((...((((........)).))...))))))).)))))))))))))..)))).))))).

***********************************************************TTTTTTGCTGGAACATTTCTGG********** SY-1-m0015-3p 8

-----------------------------------------------------------TTTTTTGCTGGAACATTTCTGG---------- t0028725 6

------------------------------------------------------------TTTTTGCTGGAACATTTCTGGT--------- t0081028 2

SY-1-m0016 chr26:34058289:34058367:+ 79(nt) -37.00(kcal/mol)

GGGAGGGGCGTAGAGAAGCACTGGGGGAAAGTCTTAGAAGTAAGATGCTGCCCCTTGATGCTTGTCTTTATCCCTTTCA SY-1-m0016 99

(..((((..(.(((.((((((.(((((...((((((....))))))....))))).).))))).))).)..))))..).

**********TAGAGAAGCACTGGGGGAAAGT*********************************************** SY-1-m0016-5p 99

----------TAGAGAAGCACTGGGGGA--------------------------------------------------- t0090438 2

----------TAGAGAAGCACTGGGGGAA-------------------------------------------------- t0021962 8

----------TAGAGAAGCACTGGGGGAAA------------------------------------------------- t0027555 6

----------TAGAGAAGCACTGGGGGAAAG------------------------------------------------ t0015692 13

----------TAGAGAAGCACTGGGGGAAAGT----------------------------------------------- t0004648 68

----------TAGAGAAGCACTGGGGGAAAGTC---------------------------------------------- t0168382 1

----------TAGAGAAGCACTGGGGGAAAGTCT--------------------------------------------- t0302951 1

SY-1-m0017 chr26:24439300:24439375:- 76(nt) -30.74(kcal/mol)

CCTACCAATCTCGACCGGACCTCGACCGGCTCGTCTATATTGCCAATCGACTCGGCGTGGCGTCGGTCGTGGTAGA SY-1-m0017 5

.((((((....(((((((.((.((.(((..(((..............)))..))))).))..))))))))))))).

**********TCGACCGGACCTCGACCGGCTCG******************************************* SY-1-m0017-5p 5

----------TCGACCGGACCTCGACCGGCTCG------------------------------------------- t0032119 5

SY-1-m0018 chr5:61789475:61789550:- 76(nt) -25.12(kcal/mol)

TGAGCAAGAGTAAGGAAAAGGCTTGTTAGGAGACCTCAGGCTCCTCCTAACTCAGCCCTTCCTTTCCAGTGGCAGA SY-1-m0018 10

...((......((((((..((((.((((((((...........))))))))..)))).)))))).......))...

**********TAAGGAAAAGGCTTGTTAGG********************************************** SY-1-m0018-5p 10

--------AGTAAGGAAAAGGCTTGTTAGG---------------------------------------------- t0057829 2

---------GTAAGGAAAAGGCTTGTTAGG---------------------------------------------- t0209251 1

---------GTAAGGAAAAGGCTTGTTAGGA--------------------------------------------- t0189269 1

----------TAAGGAAAAGGCTTGTTAG----------------------------------------------- t0104796 1

----------TAAGGAAAAGGCTTGTTAGG---------------------------------------------- t0048268 3

----------TAAGGAAAAGGCTTGTTAGGA--------------------------------------------- t0085286 2

SY-1-m0019 chr7:16299260:16299332:- 73(nt) -33.30(kcal/mol)

GCCCTGGGGTTAGGGGGAGCGGTTGGAGGACTGGGGGCTCAGCCTTCCTACCTCTCTCCGTAGGAGCCTGGTG SY-1-m0019 7

(((...((((((.((((((.(((.(((((.((((....)))).))))).))).)))))).))...))))))).

**********TAGGGGGAGCGGTTGGAGGACT***************************************** SY-1-m0019-5p 7

---------TTAGGGGGAGCGGTTGGAGGAC------------------------------------------ t0262575 1

----------TAGGGGGAGCGGTTGGAGGAC------------------------------------------ t0052676 3

----------TAGGGGGAGCGGTTGGAGGACT----------------------------------------- t0046222 3

SY-1-m0020 chr8:9359007:9359088:- 82(nt) -27.60(kcal/mol)

AGCCCCATTCTTGGCATTCACCGCGTGCCTTAATTGTATGGACATTTAAATCAAGGTCCGCTGTGAACACGGAGAGAGAGGC SY-1-m0020 23

.(((.(.(((((.(..(((((.(((..((((.(((..((....))...))).))))..))).)))))..).))))).).)))

**************************************************TCAAGGTCCGCTGTGAACACGG********** SY-1-m0020-3p 23

--------------------------------------------------TCAAGGTCCGCTGTGAACACG----------- t0263485 1

--------------------------------------------------TCAAGGTCCGCTGTGAACACGG---------- t0012421 18

---------------------------------------------------CAAGGTCCGCTGTGAACACGG---------- t0078957 2

---------------------------------------------------CAAGGTCCGCTGTGAACACGGA--------- t0083293 2

SY-1-m0021 chrUn.004.245:10867:10949:+ 83(nt) -24.72(kcal/mol)

AGTCATTTTGAGAGGTAAAAAATTGATTTGACTAGTTCTTTAACACATCTAGCAAATCATTTTTTACTCTCCAAAAAGAACTC SY-1-m0021 225

..((.(((((.((((((((((((.((((((.((((.............))))))))))))))))))).)))))))).))....

**********AGAGGTAAAAAATTGATTTGACT************************************************** SY-1-m0021-5p 208

****************************************************CAAATCATTTTTTACTCTCCA********** SY-1-m0021-3p 16

----------AGAGGTAAAAAATTGATTTG----------------------------------------------------- t0043627 3

----------AGAGGTAAAAAATTGATTTGA---------------------------------------------------- t0012284 19

----------AGAGGTAAAAAATTGATTTGAC--------------------------------------------------- t0012866 17

----------AGAGGTAAAAAATTGATTTGACT-------------------------------------------------- t0002407 161

----------AGAGGTAAAAAATTGATTTGACTAG------------------------------------------------ t0032391 5

------------AGGTAAAAAATTGATTTGACTAG------------------------------------------------ t0408010 1

-------------GGTAAAAAATTGATTTGACT-------------------------------------------------- t0477636 1

--------------GTAAAAAATTGATTTGACT-------------------------------------------------- t0358707 1

---------------TAAAAAATTGATTTGACT-------------------------------------------------- t0433272 1

--------------------------------------------------AGCAAATCATTTTTTACTCTCC----------- t0476771 1

---------------------------------------------------GCAAATCATTTTTTACTCTCCA---------- t0073936 2

----------------------------------------------------CAAATCATTTTTTACTCTCC----------- t0033858 5

----------------------------------------------------CAAATCATTTTTTACTCTCCA---------- t0022199 8

SY-1-m0022 chrX:21829425:21829509:- 85(nt) -32.80(kcal/mol)

ATGAATGCGAAAACCTCAGTCAGCCTTGTGGATGTATGTTCTGCAGACCTGACATCTAGAGGACTGACTGAAATTTTCACTTTCA SY-1-m0022 1408

.((((.(.(((((..((((((((((((.(((((((..(((.....)))...))))))))))).))))))))..))))).).))))

*****************************************************ATCTAGAGGACTGACTGAAATT********** SY-1-m0022-3p 1408

--------------------------------------------------GACATCTAGAGGACTGACTGA-------------- t0131710 1

--------------------------------------------------GACATCTAGAGGACTGACTGAAA------------ t0115103 1

---------------------------------------------------ACATCTAGAGGACTGACTGAAA------------ t0465258 1

----------------------------------------------------CATCTAGAGGACTGACTGA-------------- t0002508 153

----------------------------------------------------CATCTAGAGGACTGACTGAA------------- t0004713 66

----------------------------------------------------CATCTAGAGGACTGACTGAAA------------ t0001541 271

----------------------------------------------------CATCTAGAGGACTGACTGAAAT----------- t0001529 274

----------------------------------------------------CATCTAGAGGACTGACTGAAATT---------- t0012448 18

-----------------------------------------------------ATCTAGAGGACTGACTGA-------------- t0012099 19

-----------------------------------------------------ATCTAGAGGACTGACTGAA------------- t0013606 16

-----------------------------------------------------ATCTAGAGGACTGACTGAAA------------ t0003537 97

-----------------------------------------------------ATCTAGAGGACTGACTGAAAT----------- t0002236 176

-----------------------------------------------------ATCTAGAGGACTGACTGAAATT---------- t0001524 275

-----------------------------------------------------ATCTAGAGGACTGACTGAAATTT--------- t0022504 8

------------------------------------------------------TCTAGAGGACTGACTGAAAT----------- t0024268 7

------------------------------------------------------TCTAGAGGACTGACTGAAATT---------- t0010429 23

------------------------------------------------------TCTAGAGGACTGACTGAAATTT--------- t0369084 1

--------------------------------------------------------TAGAGGACTGACTGAAATT---------- t0290243 1
