# Supplementary material for: Identification of Conserved and Novel microRNAs in Cashmere Goat Skin by Deep Sequencing
Source: PLoS One. 2012 Dec 7;7(12):e50001. doi: 10.1371/journal.pone.0050001 (PMC3517574; doi:10.1371/journal.pone.0050001)
Supplement: Information S5 — Submitted data. Description of data: We have submitted data in MIAME-compliant format in Gene Expression Omnibus (NCBI). (DOC) [file pone.0050001.s010.doc]

**Supporting information S5:** Submitted data

We have submitted data in MIAME-compliant format in Gene Expression Omnibus (NCBI).

Our records have been approved and assigned GEO accession numbers as appended below:

[http://www.ncbi.nlm.nih.gov/geo/query/acc.cgi?acc=GSE36052](http://www.ncbi.nlm.nih.gov/geo/query/acc.cgi?acc=GSE36052 )

The Series record GSE36052 provides access to all of the data
